# Lipidomics profiles of human spermatozoa: insights into capacitation and acrosome reaction using UPLC-MS-based approach

**DOI:** 10.3389/fendo.2023.1273878

**Published:** 2023-11-07

**Authors:** Xiaohong Cheng, Haifeng Xie, Yuping Xiong, Peibei Sun, Yamei Xue, Kun Li

**Affiliations:** ^1^ School of Pharmacy, Hangzhou Medical College, Hangzhou, China; ^2^ School of Basic Medical Sciences and Forensic Medicine, Hangzhou Medical College, Hangzhou, Zhejiang, China; ^3^ Reproductive Medicine Center, Department of Obstetrics and Gynecology, Sir Run Run Shaw Hospital, College of Medicine, Zhejiang University, Hangzhou, Zhejiang, China; ^4^ Zhejiang Provincial Laboratory of Experimental Animal’s & Nonclinical Laboratory Studies, Hangzhou Medical College, Hangzhou, Zhejiang, China

**Keywords:** lipidomics, spermatozoa, capacitation, acrosome reaction, male reproduction, sperm function, 20-carboxy-LTB4, 2-oxo-4-methylthio-butanoic acid

## Abstract

**Introduction:**

Lipidomics elucidates the roles of lipids in both physiological and pathological processes, intersecting with many diseases and cellular functions. The maintenance of lipid homeostasis, essential for cell health, significantly influences the survival, maturation, and functionality of sperm during fertilization. While capacitation and the acrosome reaction, key processes before fertilization, involve substantial lipidomic alterations, a comprehensive understanding of the changes in human spermatozoa's lipidomic profiles during these processes remains unknown. This study aims to explicate global lipidomic changes during capacitation and the acrosome reaction in human sperm, employing an untargeted lipidomic strategy using ultra-performance liquid chromatography-mass spectrometry (UPLC-MS).

**Methods:**

Twelve semen specimens, exceeding the WHO reference values for semen parameters, were collected. After discontinuous density gradient separation, sperm concentration was adjusted to 2 x 10^6^ cells/ml and divided into three groups: uncapacitated, capacitated, and acrosome-reacted. UPLC-MS analysis was performed after lipid extraction from these groups. Spectral peak alignment and statistical analysis, using unsupervised principal component analysis (PCA), bidirectional orthogonal partial least squares discriminant analysis (O2PLS-DA) analysis, and supervised partial least-squares-latent structure discriminate analysis (PLS-DA), were employed to identify the most discriminative lipids.

**Results:**

The 1176 lipid peaks overlapped across the twelve individuals in the uncapacitated, capacitated, and acrosome-reacted groups: 1180 peaks between the uncapacitated and capacitated groups, 1184 peaks between the uncapacitated and acrosome-reacted groups, and 1178 peaks between the capacitated and acrosome-reacted groups. The count of overlapping peaks varied among individuals, ranging from 739 to 963 across sperm samples. Moreover, 137 lipids had VIP values > 1.0 and twenty-two lipids had VIP > 1.5, based on the O2PLS-DA model. Furthermore, the identified twelve lipids encompassed increases in PI 44:10, LPS 20:4, LPA 20:5, and LPE 20:4, and decreases in 16-phenyl-tetranor-PGE2, PC 40:6, PS 35:4, PA 29:1, 20-carboxy-LTB4, and 2-oxo-4-methylthio-butanoic acid.

**Discussion:**

This study has been the first time to investigate the lipidomics profiles associated with acrosome reaction and capacitation in human sperm, utilizing UPLC-MS in conjunction with multivariate data analysis. These findings corroborate earlier discoveries on lipids during the acrosome reaction and unveil new metabolites. Furthermore, this research highlights the effective utility of UPLC-MS-based lipidomics for exploring diverse physiological states in sperm. This study offers novel insights into lipidomic changes associated with capacitation and the acrosome reaction in human sperm, which are closely related to male reproduction.

## Introduction

Lipidomics involves the comprehensive analysis and characterization of lipids and their systemic-level interactions (1). Lipidomics elucidates the physiological and pathological roles of lipid molecules across cellular, tissue, and organ levels and their interplay with various diseases via metabolic enzymes and signaling pathways. Lipidomics augments our understanding of membrane lipid regulation, encompassing elements such as fluidity, fusion, lipid rafts, cytoskeleton, kinases, and membrane proteins (2). Additionally, lipidomics has utility in biomarker screening, clinical trials (3, 4), and disease prognosis (5). Lipids play crucial roles in energy storage, cellular structure, and signaling, and maintaining lipid homeostasis is essential for overall health while lipid defects can contribute to pathogenesis and diseases (6, 7). In the context of reproductive biology, lipids are crucial for sperm survival, maturation, and proper functioning during fertilization (8, 9). Phospholipids, including phosphatidylcholine, phosphatidylethanolamine, phosphatidylinositol, and phosphatidylserine, are integral components of cell membranes and modulate membrane fluidity, ion channel activation, and the functionality of membrane-binding enzymes (10). Recent research has demonstrated correlations between lipidomic alterations in asthenozoospermic men and sperm motility (11).

Capacitation and the acrosome reaction are two essential sperm functions required for fertilization. Capacitation refers to the change enabling spermatozoa to acquire fertilizing capacity during the period of spermatozoa’s exposure to the female reproductive tract (12). During capacitation, the sperm undergo the regulation from the complicated signal pathways involved in ion fluxes and kinase (13, 14). Meanwhile, the lipid composition and distribution of the sperm plasma membrane change, encompassing modifications in phospholipids, cholesterol, anionic lipids, lipid methylation, and lipid diffusion (15). When capacitated, sperm are competent to undergo the acrosome reaction. The acrosome reaction, which is essential for sperm-egg membrane fusion, involves a fusion-fission process between the plasma membrane and the outer acrosomal membrane, leading to the release of acrosomal contents (16, 17). The acrosome reaction in various species can be induced by physiological and nonphysiological substances, like progesterone and calcium ionophore 23187 (17, 18). Additionally, the acrosome reaction is regulated by different signal pathways including various phospholipases and lipid signaling molecules (17, 19, 20). Thus, capacitation and the acrosome reaction are continuous but different processes in mammalian sperm (17).

However, it remains elusive how the overall lipidomic characteristics of human spermatozoa change during capacitation and the acrosome reaction despite previous research documenting the involvement of specific lipid species in mammalian spermatozoa. This study aims to investigate lipidomic changes and differences between capacitation and the acrosome reaction in human spermatozoa using ultra-performance liquid chromatography-mass spectrometry (UPLC-MS) analysis and an untargeted strategy. Contrary to targeted studies focusing on quantifying specific compounds (21), the untargeted approach facilitates the analysis of all detectable metabolites—both known and unknown—thereby unveiling significant disparities between groups (22). Additionally, the present study utilized statistical methodologies such as unsupervised principal component analysis (PCA), supervised partial least-squares latent structure discriminate analysis (PLS-DA), and bidirectional orthogonal partial least squares (O2PLS) analysis. The result revealed the identification of twelve prominent differentiating lipids or metabolites between acrosome-reacted sperm induced by A23187 and uncapacitated as well as capacitated sperm in humans. These findings demonstrate the effectiveness of combining UPLC-MS-based lipidomics and multivariate analysis within an untargeted strategy to discern distinctive lipids across physiological states in sperm. Furthermore, they provide fresh insights into understanding lipid physiology during the acrosome reaction and capacitation in human sperm.

## Materials and methods

### Chemical reagents

The human tubal fluid (HTF) medium was prepared using previously described methods (23, 24). The HTF medium (100 ml) comprised the following components: 90.0 mM NaCl, 25.05 mM NaHCO_3_, 4.96 mM KCl, 6.98 mM MgCl_2_, 1.80 mM CaCl_2_·2H_2_O, 10.0 mM glucose, 10.0 mM sodium lactate, 0.27 mM sodium pyruvate, 1.17 mM KH_2_PO_4_, 2.0 mM HEPES, 400 mg BSA (fraction V, fatty acid-free), and 6 mg penicillin G. Sperm Wash^®^ and gradient 40% and 80% (Sperm Filter^®^) were obtained from Cryos, Denmark. Calcium ionophore A23187 was sourced from Sigma Aldrich (Steinheim, Germany). Dimethyl sulfoxide (DMSO) was acquired from Merck (Darmstadt, Germany). Methanol was procured from Sigma-Aldrich (LC-MS CHROMASOLV^®^, ≥99.9%, Fluka^®^, Steinheim, Germany). Chloroform was supplied by Juhua Group Corporation (Zhejiang, China). Acetic acid was ordered from Sinopharm Chemical Reagent Co., Ltd (Shanghai, China). HPLC-grade formic acid was sourced from Sigma-Aldrich (St Louis, MO). Distilled water was purified with a Milli-Q system (Millipore, Bedford, MA).

### Sperm preparation and study design

**Figure 1:**
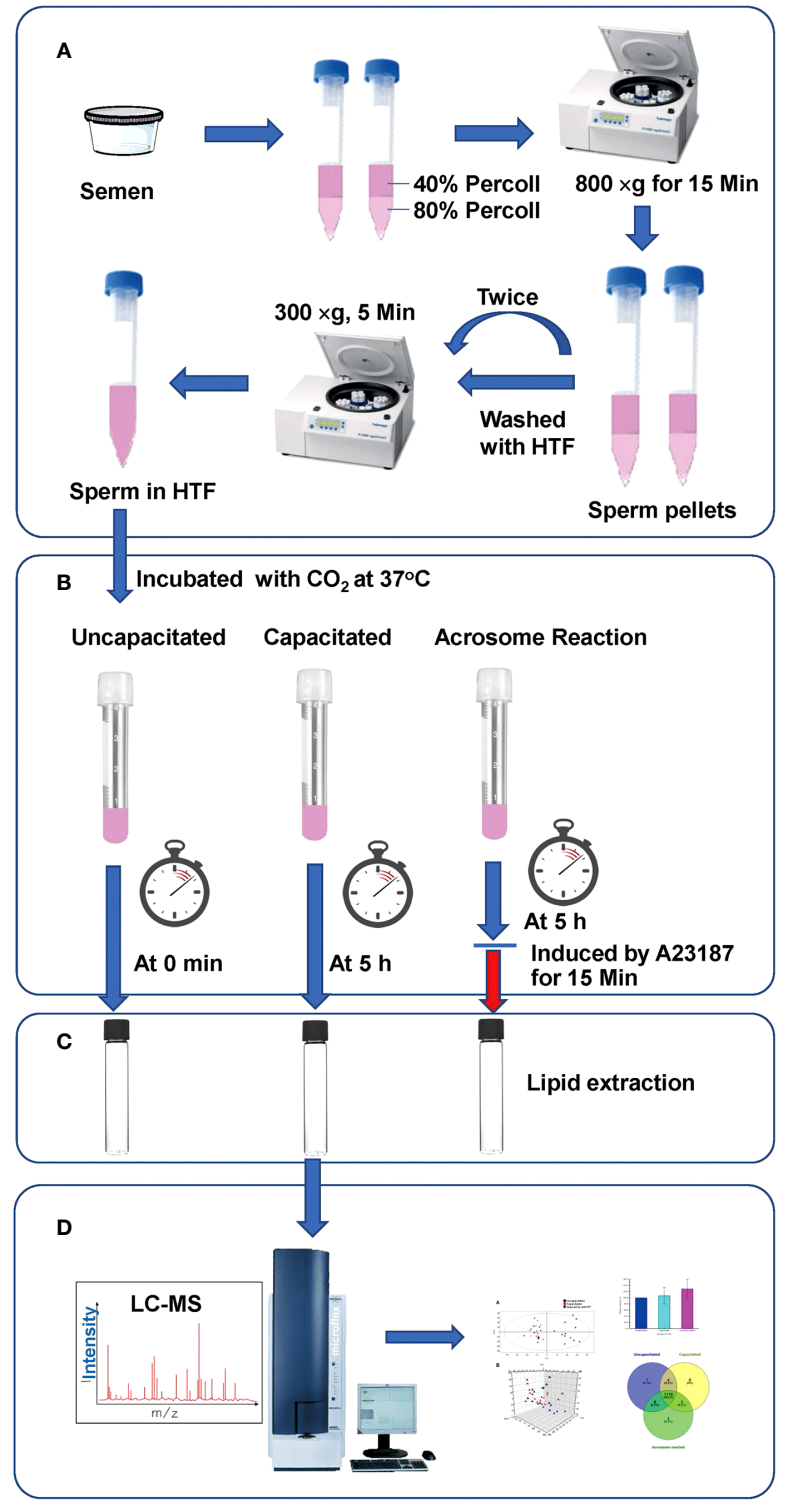
Workflow of our LC-MS-based lipidomics analysis. **(A)** Sperm Preparation; **(B)** Treatment of sperm under various conditions; **(C)** Lipid extraction; **(D)** Analysis via UPLC-MS.

This study was reviewed and approved by the Medical Ethics Committee at the Zhejiang Academy of Medical Sciences. Semen samples were donated by the healthy men who provided written informed consent. The semen samples were obtained from individuals without any reproductive disorders. The experimental design is depicted in **Figure 1**. Samples were collected in sterile plastic containers through masturbation after a period of sexual abstinence lasting 3–5 days. Semen parameters were assessed following the guidelines of the World Health Organization (25). Semen samples containing other cell types were excluded from the study. Twelve semen samples meeting the criteria for normospermia were used. Sperm preparation was carried out as previously described (26). Briefly, sperm were separated from seminal plasma, via centrifugation using discontinuous Ready-to-use gradients of 40% and 80% (Sperm Filter^®^) at 800 x g for 15 min. The pellets were washed twice in centrifuge tubes with ten times the volume of Sperm Wash^®^ by centrifugation at 300 g for 5 min. The concentrations were then adjusted to 2 x 10^6^ cells/ml using HTF medium. The sperm suspension was divided into three groups before lipid extraction (1) the uncapacitated group, sperm were kept under noncapacitated conditions, on ice without 5% CO_2_ incubation as human sperm do not undergo capacitation at temperatures below 37°C (27); (2) the capacitated group, sperm were incubated for capacitation at 37°C in 5% CO_2_ for 5 h; (3) the acrosome reacted group, sperm were incubated for capacitation at 37°C in 5% CO_2_ for5 h, and treated with 10 μM A23187 (final concentration) for 15 min.

### Lipid extraction

Lipids were extracted from sperm under various conditions following the method described (28). Sperm were washed with PBS via centrifugation at 700 x g for 8 min. After resuspending the pellets in 400 μl PBS, they were vortexed in a mixture of 1.5 ml chloroform and methanol (1:2, v/v), and incubated at room temperature for 30 min. Subsequently, 0.5 ml chloroform was introduced into the mixture, followed by the addition of 0.5 ml acetic acid (40 mM), and then centrifuged at 1000 x g for 20 min. The lower chloroform phase was collected, and the remaining suspension underwent an additional extraction with 1 ml chloroform. The collected phases were combined into a single vial and dried using a nitrogen stream. The capped vials were stored at -80°C until lipid analysis.

### Lipidomics profiles acquisition

The lipidomic characteristics of all extracted sperm were initially analyzed in the same batch using UPLC−MS based on the mass-to-charge ratio (m/z) and retention time (RT) (29). A reversed-phase analysis was performed using a Waters ACQUITY UPLC system (Waters Corporation, USA) equipped with an ACQUITY UPLC BEH C18 analytical column (particle size 1.7 μm, pore size 130 Å, dimensions 2.1 mm × 100 mm). Mobile phases A and B consisted of water/formic acid (99.9:0.1, v/v) and methanol/formic acid (99.9:0.1, v/v), respectively. The column was maintained at 50°C, and elution followed a gradient over 25 min: 0-0.5 min with 60% mobile phase B, 0.5-4 min increased to 80% B, 4-10 min increased to 98% B and held for 7 min, 17-18 min increased to 100% B and held for 1 min, 19-19.5 min mobile phase B was decreased to 3% and held for another 5.5 min for column re-equilibration. The flow rate was set at 0.3 ml/min, and the sample manager temperature was set at 4°C, with 2 μl of the sample injected into the column. Mass spectrometry was performed using the positive ion electrospray mode of the Waters Q-TOF Premier mass spectrometer (Waters Corporation, USA). Instrumental parameters were optimized as follows: a mass scan range of 50 to 1000 m/z with an accumulation time of 0.2 s per spectrum, an MS acquisition rate of 0.3 s, an interscan delay of 0.02 s, nebulizer and drying gas consisting of high-purity nitrogen, a nitrogen drying gas flow rate of 450 l/h, and a source temperature of 110°C. In the positive ion mode, the capillary voltage was set to 3.0 kV, the sampling cone voltage to 45.0 V, and the collision gas to Argon. MS/MS analysis was carried out with a varying collision energy of 10−50 eV, depending on the stability of each substance. The time-of-flight analyzer was used in V mode and tuned for maximum resolution (>10,000 resolving power at m/z 556.2771). The instrument was calibrated with sodium formate, and the lock mass spray for precise mass determination was set at 0.5 μg/ml leucine enkephalin at m/z 556.2771 in the positive ion mode. The quality control (QC) strategy is followed: QC samples were analyzed at intervals of eight samples during the analytical run, and the accuracy and precision of the assay method were evaluated via the QC sample analysis. All analyses were acquired via the lock spray to ensure accuracy and reproducibility, with the lock spray frequency set at 5 s, and data averaged over ten scans. Data were collected in centroid mode.

### Data processing and analysis

All data from UPLC−MS analyses underwent processing using MarkerLynx applications with Waters MassLynx software (v4.1, Waters). Lipids were identified and validated using the Progenesis QI software. Peak intensities of the samples were detected, integrated, and normalized with applications (29–33). The primary parameters were applied according to the supporting information provided by the software. The total peak area for each sample was defined as a constant of 1000. To investigate the differences between the groups, the lipidomic features were statistically analyzed using multivariate data analysis.

The resulting multivariate datasets comprised a single matrix with RT−m/z pairs for each file and were analyzed with SIMCA-P^+^ 14.0 (Umetrics AB, Sweden) using multivariate data analysis techniques to visualize lipid clustering among different groups of samples. Before statistical analysis, data were normalized, mean-centered, and Pareto-scaled (29–31). An initial unsupervised PCA was used for general clustering visualization and outlying detection. Subsequently, supervised PLS-DA and bidirectional orthogonal partial least squares (O2PLS) analysis were performed to identify lipidomic changes between different conditions contributing to the observed clustering in the PCA (29–31, 34). To prevent model overfitting, the supervised models were validated with a permutation test repeated 200 times. Lipids potentially changed between different groups were selected based on variable importance in the projection (VIP) values and the S-plot. VIP values reflect the influence of each lipid composition on group differences while the S-plot, a loading plot, visually assisted in selecting major altered lipids. Variables farthest from the origin in S-plots significantly contribute to the inter-group differences (29).

### Statistical analysis

The statistical significance of the data in different treatment groups was analyzed using SPSS 25.0 software. The one-way ANOVA test and the Mann-Whitney test were employed to evaluate the statistical significance between different groups (P < 0.05). Before analysis, normal distribution using the Shapiro-Wilk test and homogeneity of variances in the data among different groups was assessed. To identify potential lipids with differences between groups, the databases of CEU Mass Mediator (http://ceumass.eps.uspceu.es/mediator), LIPID MAPS (https://www.lipidmaps.org/), and HMDB (https://www.hmdb.ca/) were queried with the exact mass of lipids (and retention time). Additionally, Progenesis QI software (Waters) was used to validate the MS/MS profiles of the identified lipids.

## Results

### Lipidomics profiling of UPLC-MS in sperm under uncapacitated, capacitated, and acrosome-reacted conditions

**Figure 2:**
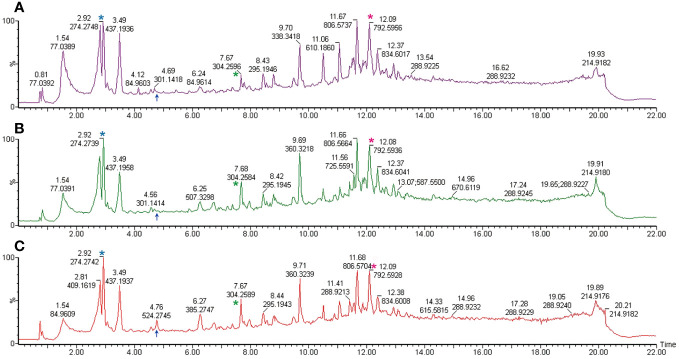
Representative total ion current chromatogram obtained from UPLC-MS. Samples are derived from the spermatozoa of one individual under differing conditions. **(A)** Uncapacitated sperm; **(B)** Capacitated sperm; **(C)** Acrosome-reacted sperm (induced by A23187). The illustration corresponds to one representative sample from each of the twelve experimental samples. The X-axis denotes the retention time, while the Y-axis represents the relative peak intensity. Common peaks are indicated with asterisks in the same colors, while different peaks are marked with blue arrows.

To delineate lipid alterations, we processed thirty-six sperm samples from twelve subjects following the experimental workflow outlined in **Figure 1**. **Figure 2** displays the base peak chromatograms of sperm in uncapacitated, capacitated, and acrosome-reacted states The lipidomic peaks observed in the three conditions were similar, indicating stable detection conditions and consistent major lipid species, such as 2.92_274.2748, 7.67_304.2596, and 12.08_792.5936. However, specific base peaks exhibited differences in intensity and presence among the different physiological conditions. For example, the base peak at 4.76_524 was absent in the uncapacitated and capacitated sperm but present in the acrosome-reacted group. Additionally, the base peak at 6.26_507.3298 exhibited higher intensity in the capacitated group compared to the uncapacitated and acrosome-reacted groups. The relative intensity of this base peak was lower in uncapacitated and capacitated sperm in comparison to acrosome-reacted sperm. These findings suggest that lipidomic profiles fluctuate based on different conditions, which can be detected through UPLC-MS analysis.

**Figure 3:**
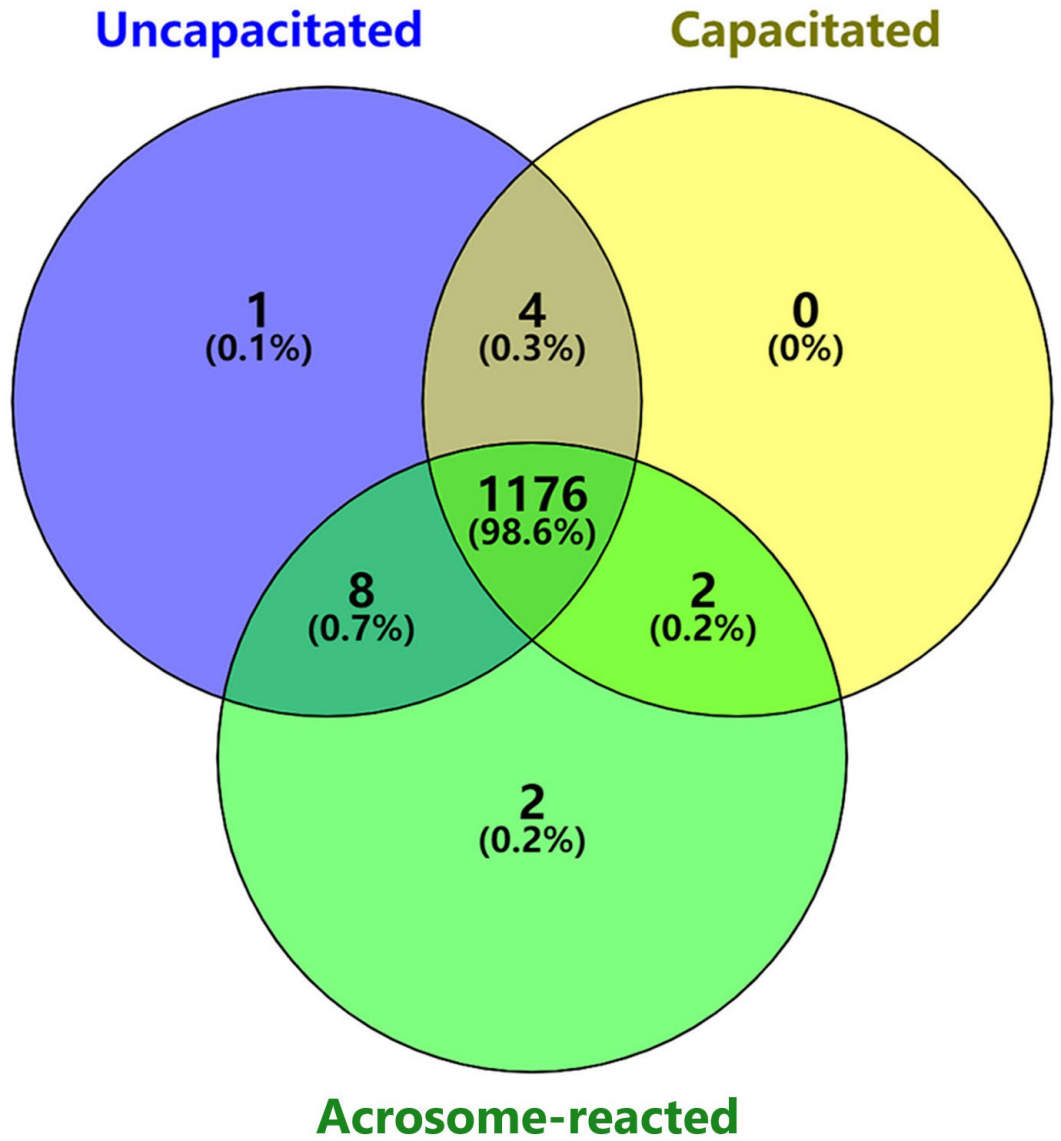
Venn diagram illustrating the number of unique and shared lipids identified among different sperm physiological states. Purple represents uncapacitated; Yellow, capacitated; Green, acrosome-reacted (A23187-induced).

Disparities in the count of lipid peaks among individuals were observed when analyzing sperm under uncapacitated, capacitated, and acrosome-reacted states (**Figure 3**). The Venn diagram illustrates the overlap of 1176 lipid peaks across the twelve individuals in the uncapacitated, capacitated, and acrosome-reacted groups. Furthermore, 1180 peaks overlapped between the uncapacitated and capacitated groups, 1184 peaks between the uncapacitated and acrosome-reacted groups, and 1178 peaks between the capacitated and acrosome-reacted groups. It is noteworthy that the count of overlapping peaks varied among individuals, ranging from 739 to 963 across sperm samples in the conditions (Refer to **Supplementary Figure 1**). Moreover, there were several peaks unique to specific groups. These results indicate that the lipid composition in sperm is influenced by different states and exhibits inter-individual variability. Untargeted studies can provide valuable information about global lipid profiles in individuals.

### Acquisition and validation of UPLC-MS data through multivariate analysis models

**Figure 4:**
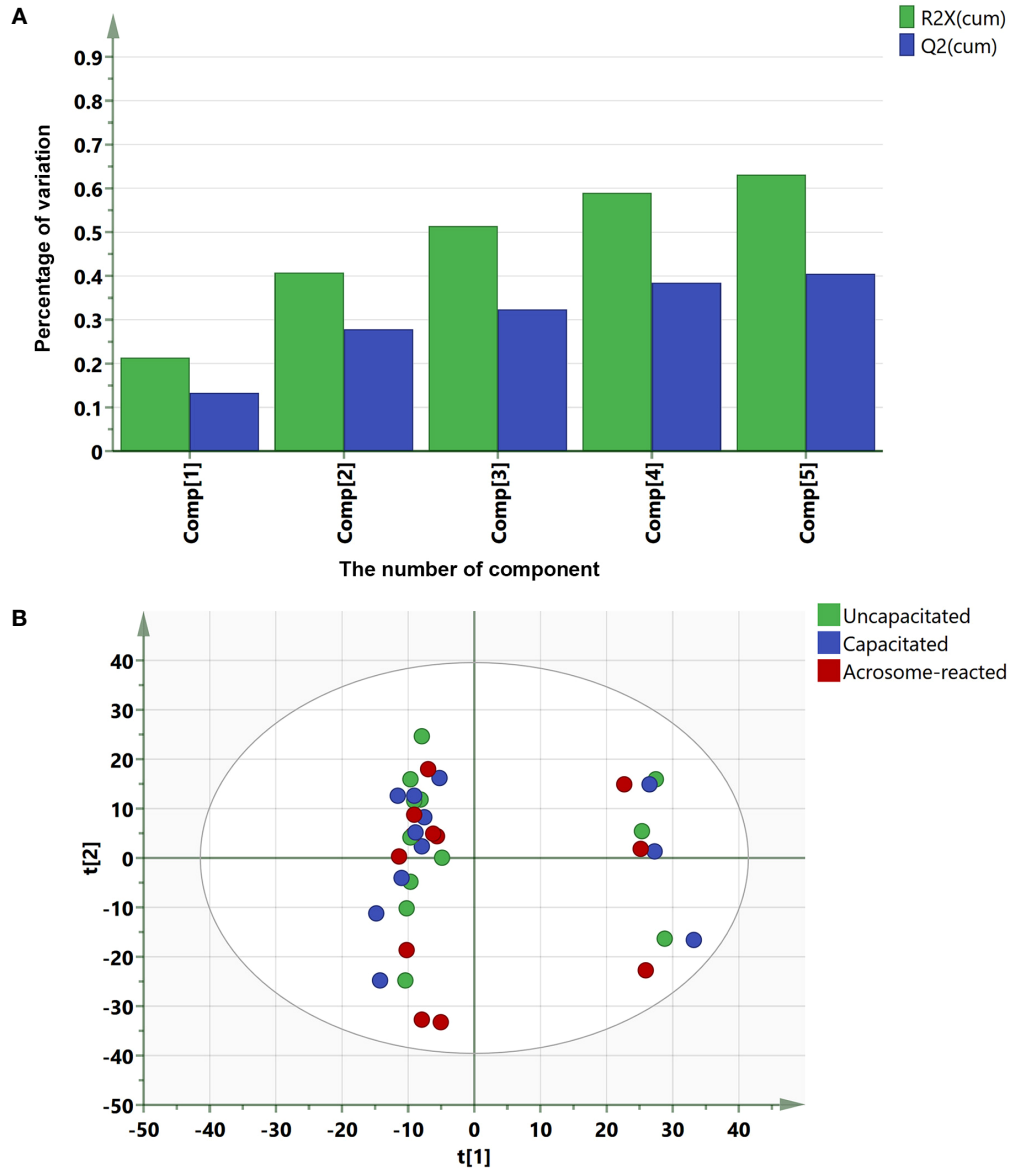
Summary of PCA fit and scores plot based on UPLC-MS data. **(A)** PCA fit summary; **(B)** Corresponding scores plot. Different circles represent different samples from twelve individuals. Blue, uncapacitated; Green, capacitated; Red, Acrosome-reacted.

To identify lipids or metabolites, we analyzed 1193 peaks in sperm under three different treatment conditions, uncapacitated, capacitated, and acrosome-reacted, using UPLC-MS. To simplify data interpretation without bias, we employed unsupervised statistical analysis, specifically PCA for the three groups. The PCA yielded five principal components (PCs), and the scatter score plot comparing PC1 and PC2 did not reveal any outliers among the uncapacitated, capacitated, and acrosome-reacted groups (**Figure 4B**). The PCA model explained 63.2% of the variance (R^2^X) and predicted 40.5% (Q^2^) of the variance based on cross-validation (**Figure 4A**).

**Figure 5:**
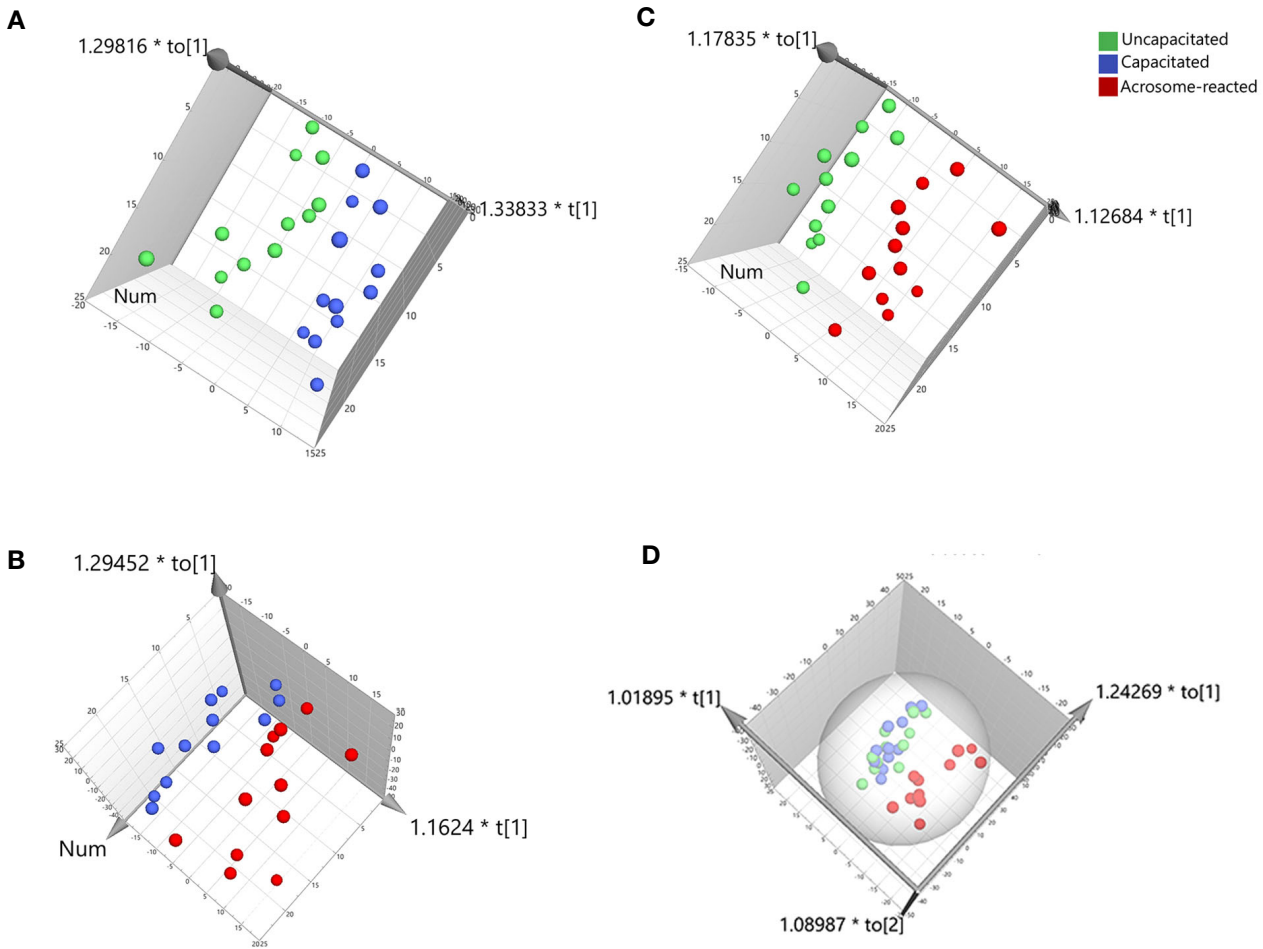
PLS-DA score plot derived from UPLC-MS data in the positive ion mode. **(A)** Comparison between Uncapacitated and Capacitated states; **(B)** Comparison between Uncapacitated and A23187-induced Acrosome-reacted states; **(C)** Comparison between Capacitated and Acrosome-reacted states; **(D)** Consolidated plot of **(A–C)**.

**Figure 6:**
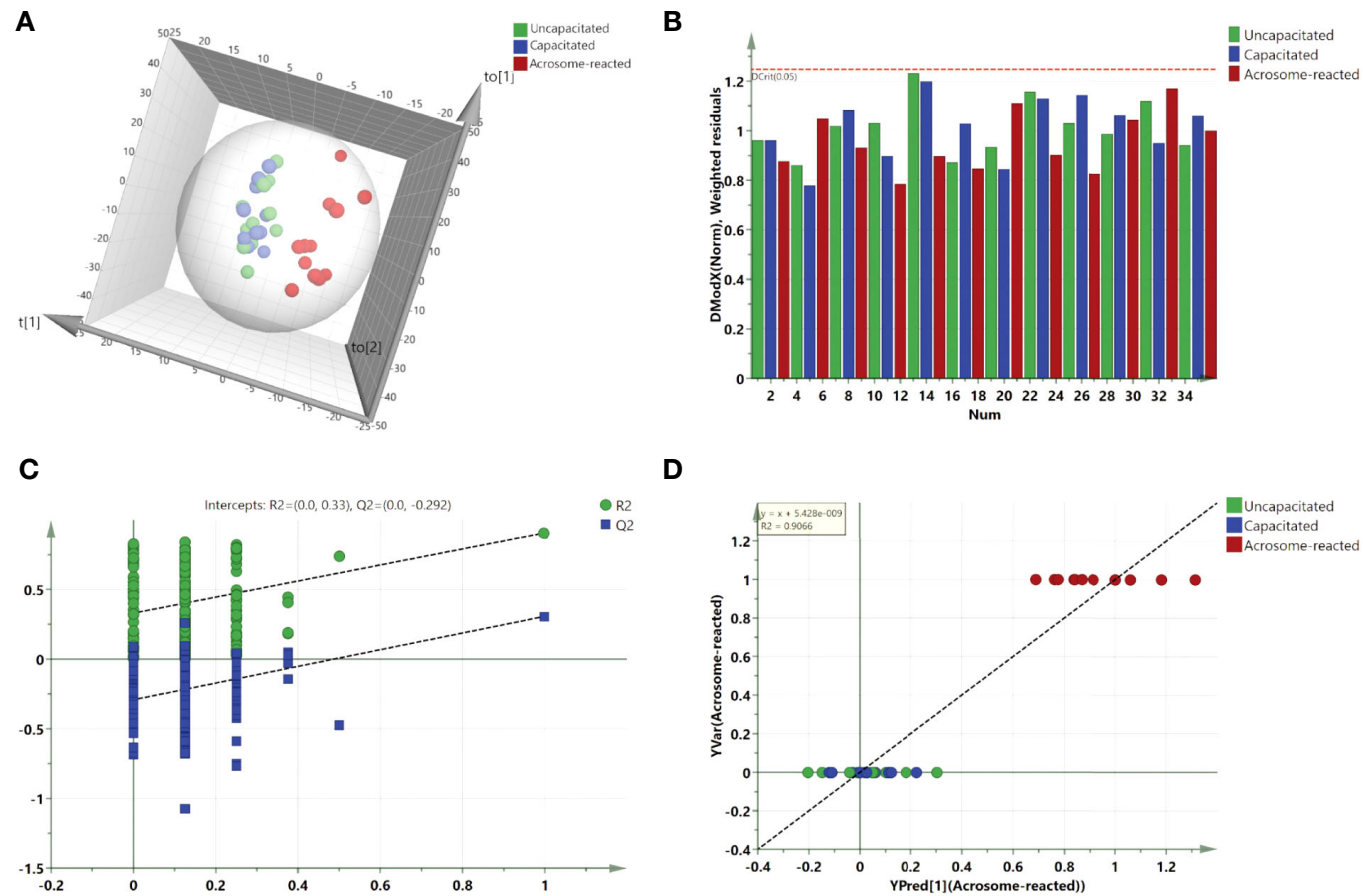
Validation and O2PLS-DA scores plot derived from UPLC-MS data in the positive ion mode. **(A)** O2PLS-DA scores plot comparing Uncapacitated and Capacitated states with Acrosome-reacted samples; **(B)** DModX plot of the O2PLS-DA model, demonstrating that the observations fall within the normal range when considering the correlation structure of the variables; **(C)** Validation plot of the combined model, based on 200 permutation tests. The R^2^ value (green line) signifies explained variance, and the Q^2^ value (blue line) denotes the predictive ability of the model. Lower calculated R^2^ and Q^2^ values than the original, and a Q^2^ intersecting the vertical axis below zero, confirm the model’s validity. **(D)** Y prediction scatter plot of all sperm samples as per the O2PLS-DA model.

For further class differentiation, supervised PLS-DA score plots were generated for the thirty-six samples. The results showed a clear separation between the uncapacitated and capacitated groups (**Figure 5A**), the uncapacitated group and the A23187-induced acrosome-reacted group (**Figure 5B**), and the capacitated group and the acrosome-reacted group (**Figure 5C**). Notably, no significant difference was observed between the uncapacitated and capacitated groups (**Figure 5D**). Subsequently, we developed an orthogonal partial least squares discriminant analysis (O2PLS-DA) model (1 + 5 + 0) to amplify the separation between the uncapacitated and capacitated groups as well as the A23187-induced acrosome-reacted group. The O2PLS-DA model (1 + 5 + 0) exhibited clear separation among the uncapacitated, capacitated, and acrosome-reacted groups (**Figure 6A**) with validated modeling (R^2^X, 65.3%; R^2^Y, 46.4%; and Q^2^, 0.155). The Distance to Model in the X space (DModX) plot of the O2PLS-DA model indicated that observations had values below 2.0, signifying their classification as standard observations in terms of variable correlation structure (**Figure 6B**). The predictive ability of the O2PLS-DA model was validated using a permutation test, which confirmed that the model did not overfit and outperformed 200 different permuted models (intercept of R^2^: 0.33; intercept of Q^2^: -0.292) (**Figure 6C**). The scatter plot of Y predictions showed a regression line with an R^2^ value of 0.9066, indicating a good model fit (**Figure 6D**).

### Identification of distinct lipids between acrosome-reacted group and uncapacitated/capacitated group

**Figure 7:**
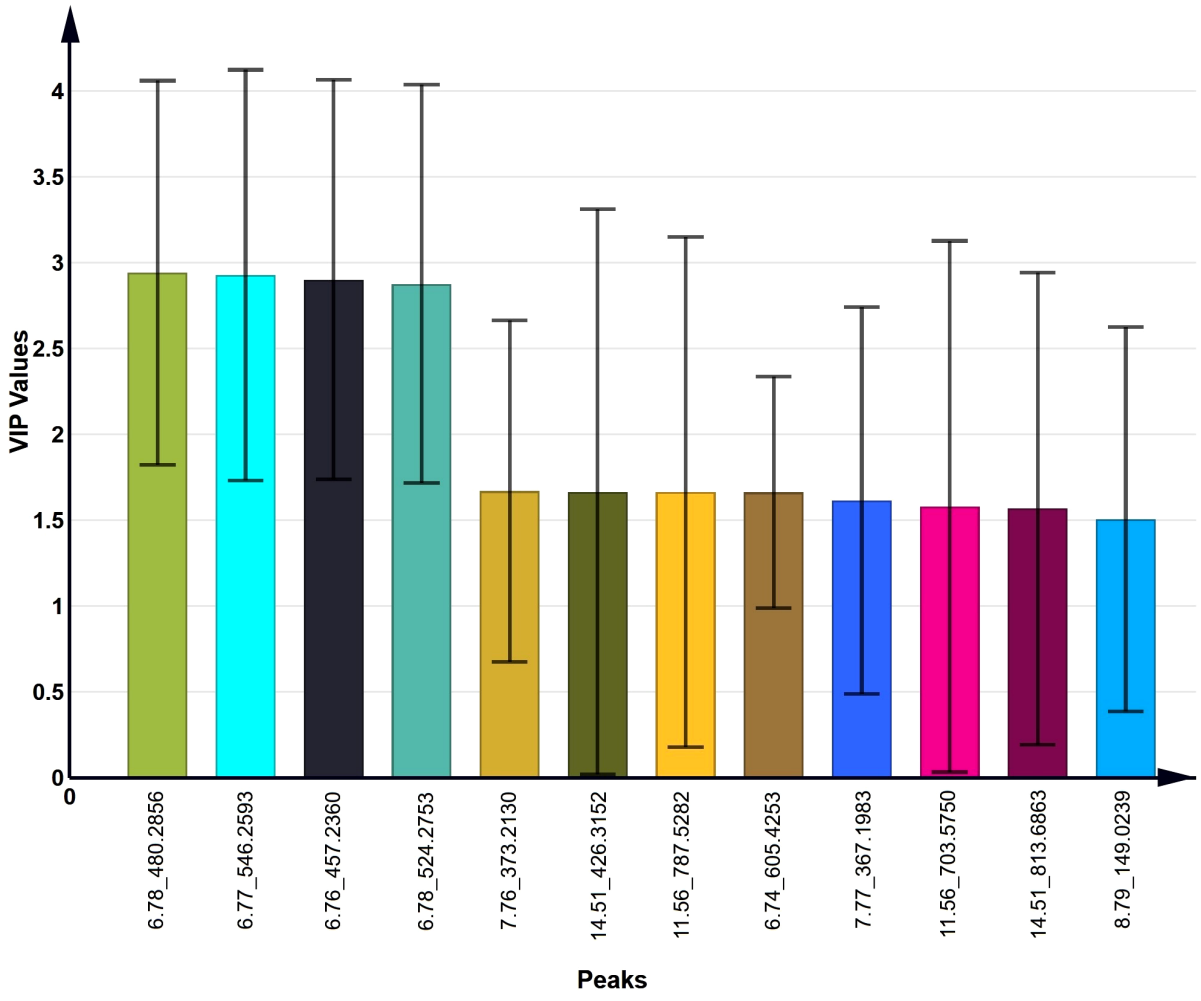
The top 12 VIP values of candidate variables obtained from the O2PLS-DA model.

**Figure 8:**
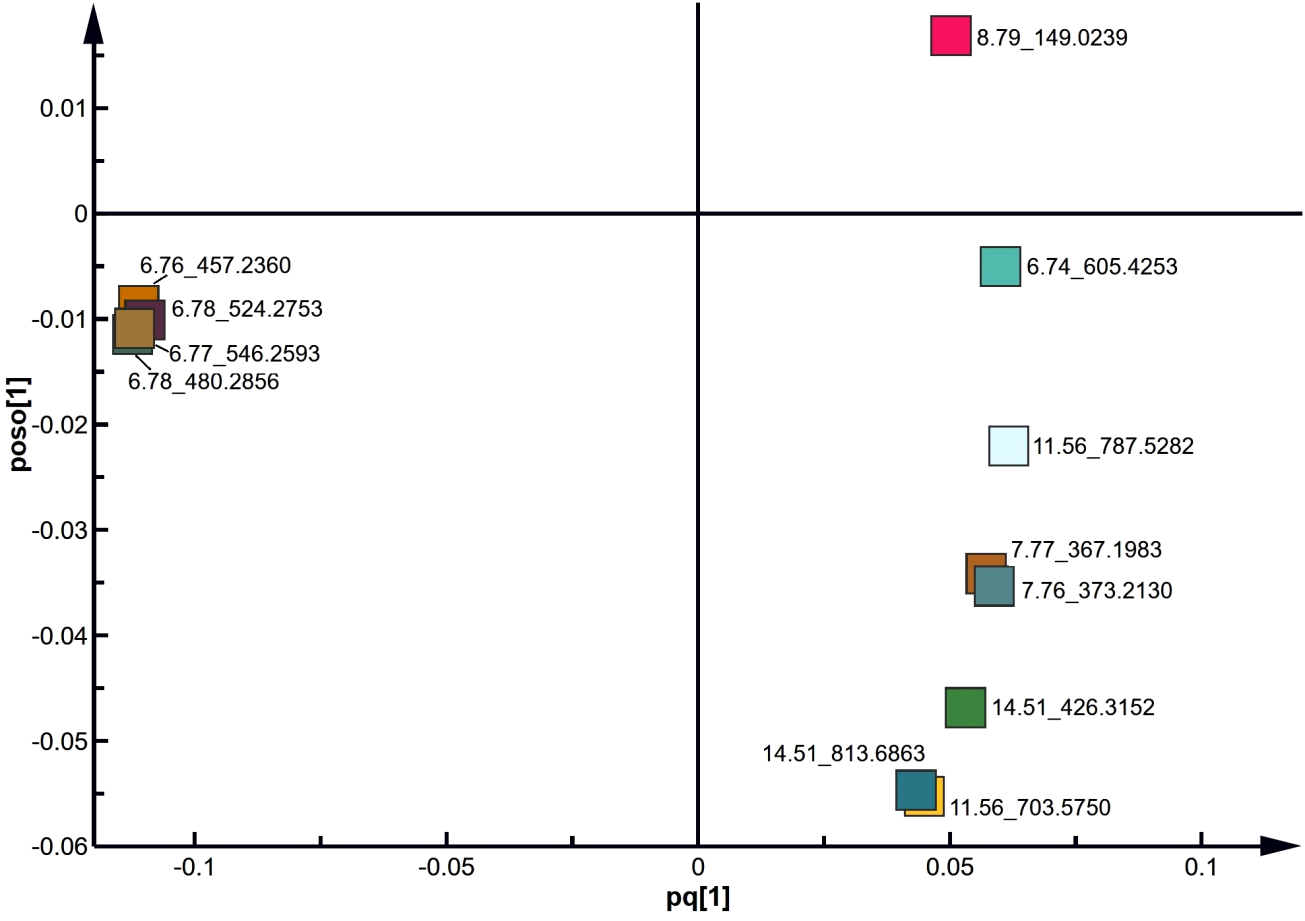
S plot derived from the O2PLS-DA model using UPLC-MS data.

To identify the lipids contributing to group differentiation and elect significant candidate variables, we initially evaluated the VIP scores of variables (per the rationale: VIP-values >1 indicate importance; < 0.5, unimportance; and the range between 1 and 0.5, uncertainty in the importance, depending on the data set size) (**Figure 7**) and identified significant regions in the S-plot of the O2PLS-DA model (**Figure 8**). Based on VIP scores exceeding 1.5 and statistical significance, we selected and identified the top twelve candidate variables, which are categorized and presented in **Table 1**. These candidate variables included diacylglycerophosphoinositols (PI), monoacylglycerophosphoserines (LPS), monoacylglycerophosphates (LPA), oxidized glycerophosphoethanolamines (LPE), fatty acyls (FA), glycerophosphocholines (PC), glycerophosphoserines (PS), glycerophosphates (PA), and ceramide phosphocholines (SM, sphingomyelins). These findings suggest that these lipid metabolites play roles in biological functions during the acrosome reaction in human sperm.

**Table 1:** The predominant discrimination metabolites in human sperm under the different physiological status.

No.	Rt_M/Z	Adducts	Identified Results	Common Name	Systematic Name	Uncapacitated	Capacitated	Acrosome-Reacted
1	6.78_480.2856	[M+2H]^+^	PI 44:10	PI (22:4 (7Z,10Z,13Z,16Z)/22:6 (4Z,7Z,10Z,13Z,16Z,19Z))	1-(7Z,10Z,13Z,16Z-docosatetraenoyl)-2-(4Z,7Z,10Z,13Z,16Z,19Z-docosahexaenoyl)-glycero-3-phospho-(1'-myo-inositol)	0.01 ± 0.04	0.03 ± 0.07	5.39 ± 3.89^ac,bc^
2	6.77_546.2593	[M+H]^+^	LPS 20:4	PS (20:4 (5Z,8Z,11Z,14Z)/0:0)	1-(5Z,8Z,11Z,14Z-eicosatetraenoyl)-sn-glycero-3-phosphoserine	0.01 ± 0.02	0.05 ± 0.13	22.29 ± 16.51 ^ac, bc^
3	6.76_457.2360	[M+H]^+^	LPA 20:5	LysoPA (20:5 (5Z,8Z,11Z,14Z,17Z) /0:0)	1-(5Z,8Z,11Z,14Z,17Z-eicosapentaenoyl)-glycero-3-phosphate	0.00 ± 0.00	0.00 ± 0.00	2.14 ± 1.65 ^ac, bc^
4	6.78_524.2753	[M+Na]^+^	LPE 20:4	LysoPE (0:0/20:4 (8Z,11Z,14Z,17Z))	2-(8Z,11Z,14Z,17Z-eicosatetraenoyl)-sn-glycero-3-phosphoethanolamine	0.26 ± 0.26	0.49 ± 1.00	113.17 ± 87.42 ^ac, bc^
5	7.76_373.2130	[M+H]^+^	FA 22:8;O3	16-phenyl-tetranor-PGE2	9-oxo-11R,15S-dihydroxy-16-phenyl-17,18,19,20-tetranor-5Z,13E-prostadienoic acid	1.38 ± 0.75	1.01 ± 0.53	0.60 ± 0.37 ^ac^
6	14.51_426.3152	[M+H+NH_4_]^+^	PC 40:6	PC (18:0/22:6 (9Z,11Z,13Z,15Z,17Z,19))	1-octadecanoyl-2-(9Z,11Z,13Z,15Z,17Z,19-docosahexaenoyl)-sn-glycero-3-phosphocholine	6.52 ± 1.51	6.34 ± 1.50	5.19 ± 1.84^bd^
7	11.56_787.5282	[M+NH_4_]^+^	PS 35:4	PS (22:4 (7Z,10Z,13Z,16Z)/13:0)	1-(7Z,10Z,13Z,16Z-docosatetraenoyl)-2-tridecanoyl-glycero-3-phosphoserine	10.63 ± 3.50	11.15 ± 2.88	7.90 ± 2.74^bd^
8	6.74_605.4253	[M+H]^+^	PA 29:1	PA (12:0/17:1(9Z))	1-dodecanoyl-2-(9Z-heptadecenoyl)-glycero-3-phosphate	4.19 ± 1.65	4.06 ± 0.96	3.08 ± 1.00 ^ad^
9	7.77_367.1983	[M+NH_4_]^+^	FA 20:5;O4	20-carboxy-LTB4	5S,12R-dihydroxy-6Z,8E,10E,14Z-eicosatetraene-1,20-dioic acid	3.63 ± 1.62	2.38 ± 1.56^ad^	1.50 ± 0.94 ^ac^
10	11.56_703.5750	[M+H]^+^	SM 34:1;O2	SM (d16:1/18:0)	N-(octadecanoyl)-hexadecasphing-4-enine-1-phosphocholine (Ceramide phosphocholines (sphingomyelins)	50.12 ± 10.66	49.24 ± 11.64	42.22 ± 14.05
11	14.51_813.6863	[M+H]^+^	SM 42:2;O2	SM (d18:1/24:1(15Z))	N-(15Z-tetracosenoyl)-sphing-4-enine-1-phosphocholine	18.08 ± 4.73	16.82 ± 4.46	14.61 ± 5.45
12	8.79_149.0239	[M+H]^+^	FA	2-oxo-4-methylthio-butanoic acid	2-oxo-4-methylthio-butanoic acid	10.96 ± 2.03	11.79 ± 2.64	9.48 ± 2.25^bd^

All data are presented as mean ± SD of peak intensities. The statistical significance was analyzed using SPSS 25.0 software. The one-way ANOVA test and the Mann-Whitney test were performed. Before analysis, normal distribution using the Shapiro-Wilk test and homogeneity of variances in the data among different groups was assessed. P values < 0.05 were considered statistically significant. a denotes comparison with the uncapacitated group, b denotes comparison with the capacitated group, c indicates P < 0.01, and d indicates P < 0.05. Lipids are formally identified by standard samples or published data. lipids putatively annotated by library searching.

RT, retention time; m/z, mass-to-charge ratio; VIP, variable importance in the project values; PI, diacylglycerophosphoinositols; LPS, monoacylglycerophosphoserines; LPA, monoacylglycerophosphates; LPE, oxidized glycerophosphoethanolamines; FA, fatty acyls; PC, glycerophosphocholines; PS, glycerophosphoserines; PA, glycerophosphates; SM, ceramide phosphocholines (sphingomyelins).

## Discussion

To the best of our knowledge, this study has been the first time to investigate the lipid profiles associated with acrosome reaction and capacitation in human sperm, utilizing UPLC-MS in conjunction with multivariate data analysis. It provides an in-depth analysis of metabolic lipids under various physiological states, encompassing the assessment of 1193 distinct lipids during uncapacitated, capacitated, and A23187-induced acrosome reaction states. It is noteworthy that the counts of lipid exhibited variability among individuals under different conditions (Refer to **Supplementary Figure 1**). This variability in the lipid metabolic compositions among individuals can be attributed to genetics and epigenetic variations influencing the expression and activity of enzymes and proteins involved in lipid metabolism as well as factors related to health conditions, lifestyle choices, and environmental exposures affecting lipid metabolism.

Furthermore, based on the VIP list derived from the O2PLS-DA model, 137 lipids had VIP values exceeding 1.0, and twenty-two lipids had VIP values exceeding 1.5. Among the identified lipids, twelve metabolites were categorized into three groups (**Table 1**): four lipids exhibited increased levels, six lipids showed decreased levels, and two lipids did not show statistically significant changes. These findings underscore the significant divergence in lipid composition during the A23187-induced acrosome reaction compared to uncapacitated and capacitated states in human sperm.

This study unveiled a higher level of PI 44:10 during the A23187-induced acrosome reaction (**Table 1**). The observed increase in PI within human sperm aligns with findings from a prior study employing a different analytical method (35). Additionally, the increased PI induced by A23187 aligns with reports in the platelet system (36). Polyphosphoinositides (PPIn) are critical signaling phospholipids that play a significant role in the composition of cellular membranes and signaling cascades in mammalian cells (37) and sperm development (38). The metabolism of polyphosphoinositides is closely linked to the activity of phosphatase and tensin homolog (PTEN) identified in human sperm during the acrosome reaction (39). This signaling pathway suggests a close association between the acrosome reaction and lipid phosphatases that remove phosphate groups through dephosphorylate, such as PTEN, SYNJ1, and SYNJ2 (37).

A significantly lower level of phosphatidylcholine (PC) 40:6 was observed during induction by A23187. These results align with previous research demonstrating a reduction in PC during acrosome reaction (40). The decline of PC levels in acrosome-reacted sperm may be a result of hydrolysis by phospholipase C (41) or phospholipase A2 (PLA_2_) (42), leading to the production of LysoPC. LysoPC, a byproduct of PC hydrolysis, independently modulates ionic gradients and induces depolarization of the sperm membrane (43), thereby enhancing sperm penetration rate and acrosome reaction (44). Additionally, it modifies the zona pellucida and the plasma membrane of both gametes *in vitro*, promoting gamete fusion (45). LysoPC is closely associated with sperm motility (46–48), the freeze/thaw cycle of sperm (49), obesity (50), and male infertility (51). LysoPC is often used as an inducer of the acrosome reaction (52) to treat spermatozoa in cases of male infertility because it increases fertilization rates in IVF (44). PC metabolism through the production of LysoPC is crucial for sperm function and fertilization.

Two classes of sphingomyelin (SM), a sphingolipid variant found in sperm plasma membranes, exhibited changes in human sperm subjected to A23187 induction, in comparison to uncapacitated and capacitated sperm. SM encompasses various molecular species, including relatively long saturated chains (16:0, 18:0, and 24:0), very long-chain polyunsaturated fatty acids (ranging from 24:0 to 34:0), and elongated versions of common PUFA from the n-6 or n-3 series (53). Our findings align with decreasing trends observed in a similar context in a previous investigation (54), although statistical significance was not observed in the present study. Furthermore, SM in mammalian sperm often contains very long-chain polyunsaturated fatty acids and can accelerate the acrosome reaction stimulated by progesterone, linked to PLA_2_ activity during the acrosome reaction (55).

Modifications in other phospholipids were observed in sperm subjected to A23187-induced acrosome reaction, as compared to capacitated and uncapacitated sperm. Specifically, LPS 20:4, LPA 20:5, and LPE 20:4 showed an increase, while PS 35:4 and PA 29:1 showed a decrease. The synthesis of PS, PA, and PC relates to glycerol-3-phosphate, known as the Kennedy pathway. Lipids such as LPA 20:5 may be regulated by glycerol-3-phosphate acyltransferases (GPATs), which esterify an acyl chain of glycerol-3-phosphate (G3P) to G3P at the sn-1 position, and LPA-acyltransferases (LPAATs), which esterify a second acyl chain of LPA to the sn-2 position to produce PA. The observed increase in PA in this study is consistent with a prior study (35). PA can be dephosphorylated by PA phosphatases (PAPs) to produce the lipid second messenger diacylglycerol (DAG), which can further be metabolized to produce PC, PE, PS, and triglyceride (TG). LPS, PS, and LPE change in the Lands’ cycle reaction and are associated with specific enzymes like LPEAT, LPSAT, LPIAT, and PLA_2_. On the other hand, PA may be acted upon by cytidine diphosphate (CDP)-DAG synthase to produce CDP-DAG, which can be further metabolized to produce PI (56) and affect the PI pathway. In this process, phospholipases, including the main PLA_2_ and phospholipases C (PLC), play roles in regulating lipid metabolism and lipid signaling molecules, participating in various signal pathways: PLA_2_ action on PC, PE, or PI generates lysoPC, lysoPE, or lysoPI as well as fatty acids such as arachidonic, oleic, linoleic, linolenic, or docosahexaenoic acid; phosphoinositide-specific PLC (PPI-PLC) mediated hydrolysis of phosphatidylinositol 4,5-bisphosphate generates 1,4,5-trisphosphate (IP3) and DAG; and PC-PLC hydrolysis of phosphatidylcholine yields choline phosphate and DAG (19, 20).

Furthermore, observations also indicated metabolic variations in different fatty acids, such as FA 22:8-O3 and FA 20:5-O4, during the A23187-induced acrosome reaction. FA 22:8-O3, also known as 16-phenyl-tetranor-prostaglandin E2 (PGE2), has been reported as a stable derivative of PGE2 that acts on its receptor (57). This result is consistent with previous studies indicating that prostaglandins play an important role in the sperm acrosome reaction (58). The other fatty acid, FA 20:5-O4, also known as 20-carboxy-leukotriene B4 (LTB4), has been associated with the action of calcium ionophores (59). LTB4 is biosynthesized from arachidonic acid released from membrane phospholipids through the activity of cytosolic and various types of PLA_2_s (60). LTB4 can induce the acrosome reaction when present throughout the preincubation period, suggesting that it may enhance the capacitation process rather than the acrosome reaction. Additionally, LTB4 is one of the metabolites linked to the adverse effects of environmental arsenic exposure on humans (61).

Of utmost importance, this study is the first to detect 2-oxo-4-methylthiobutanoic acid (OMBA), during the A23187-induced acrosome reaction. OMBA falls within the category of fatty acids and conjugates of lipids, as per lipidmaps.org, and serves as an intermediate in the metabolism of cysteine and methionine. OMBA has previously been associated with male infertility and arsenic exposure (61) and has been discussed as a metabolite marker in recent studies (62–64). It is proposed that OMBA’s impact on sperm viability may be mediated through methionine oxidation (65). Additionally, OMBA participates in the seven signaling pathways, according to the Kyoto Encyclopedia of Genes and Genomes (KEGG) Pathway Database, including cysteine and methionine metabolism, amino acid metabolism, glucosinolate biosynthesis, biosynthesis of plant secondary metabolites, metabolic pathways, biosynthesis of secondary metabolites, and oxo-carboxylic acid metabolism. The involvement of OMBA in diverse signaling pathways suggests its potential significance in sperm physiology. In all, this study suggests that OMBA and its associated signaling pathways may play a crucial role in the acrosome reaction, with further mechanistic investigations warranted in the future.

Furthermore, the applications of UPLC-MS in conjunction with multivariate data analysis effectively highlighted the dominant lipids involved in the A23187-induced acrosome reaction in human sperm. The untargeted lipidomics strategy provided comprehensive insights into the lipid composition of sperm in different physiological conditions, allowing for the detection, analysis, and differentiation of major compounds without bias. Compared to targeted strategies with specific aims, the untargeted strategy harnessed the full m/z information of molecular structure using UPLC-MS, surpassing previous lipid studies that employed various chromatography techniques. This study underscores the value of the untargeted lipidomics strategy for studying lipid metabolism and characterizing physiological states in sperm.

While acknowledging the significance, this study’s limitations should be recognized. The lipidomics analysis was exclusively performed with the positive model of UPLC-MS, potentially leading to the non-detection of some compounds in the neutral and negative ion model, thus bypassing the partial metabolites and signaling pathways. Additionally, the sample size of twelve individuals may be limited although this study has already yielded valuable insights into these differences in different physiological states. Therefore, future investigations, involving larger sample sizes and more diverse cohorts, may be necessary to thoroughly elucidate differences in lipid composition, validate findings, and generalize the results.

## Conclusions

This study has shed light on the variations in lipid composition among uncapacitated, capacitated, and acrosome-reacted sperm. Twelve significant lipids or metabolites were discerned between A23187-induced acrosome-reacted sperm and the uncapacitated/capacitated sperm in humans. The results corroborate earlier discoveries on lipids during the acrosome reaction and unveil a new metabolite OMBA. This investigation demonstrates the effectiveness of an untargeted approach using UPLC-MS-based lipidomics and multivariate analysis for identifying discriminating lipids in different physiological stages of sperm. Additionally, it provides novel insights into lipid metabolism during capacitation and the acrosome reaction in human sperm, which holds significance in the context of male reproduction.

## Data availability statement

The data presented in the study are deposited in the figshare repository, and can be found here https://doi.org/10.6084/m9.figshare.24223207.v2.

## Ethics statement

The studies involving humans were approved by the Medical Ethics Committee at the Zhejiang Academy of Medical Sciences. The studies were conducted in accordance with the local legislation and institutional requirements. The participants provided their written informed consent to participate in this study.

## Author contributions

XC: Conceptualization, Investigation, Writing – original draft. HX: Conceptualization, Investigation, Writing – review & editing. YuX: Investigation, Writing – review & editing. PS: Resources, Writing – review & editing. YaX: Conceptualization, Resources, Writing – review & editing. KL: Conceptualization, Data curation, Formal Analysis, Funding acquisition, Investigation, Methodology, Project administration, Resources, Software, Supervision, Validation, Visualization, Writing – original draft, Writing – review & editing.
