# Supplementary material for: Lipidomics profiles of human spermatozoa: insights into capacitation and acrosome reaction using UPLC-MS-based approach
Source: Front Endocrinol (Lausanne). 2023 Nov 7;14:1273878. doi: 10.3389/fendo.2023.1273878 (PMC10660817; doi:10.3389/fendo.2023.1273878)
Supplement: Supplementary file 2 [file Image_1.pdf]

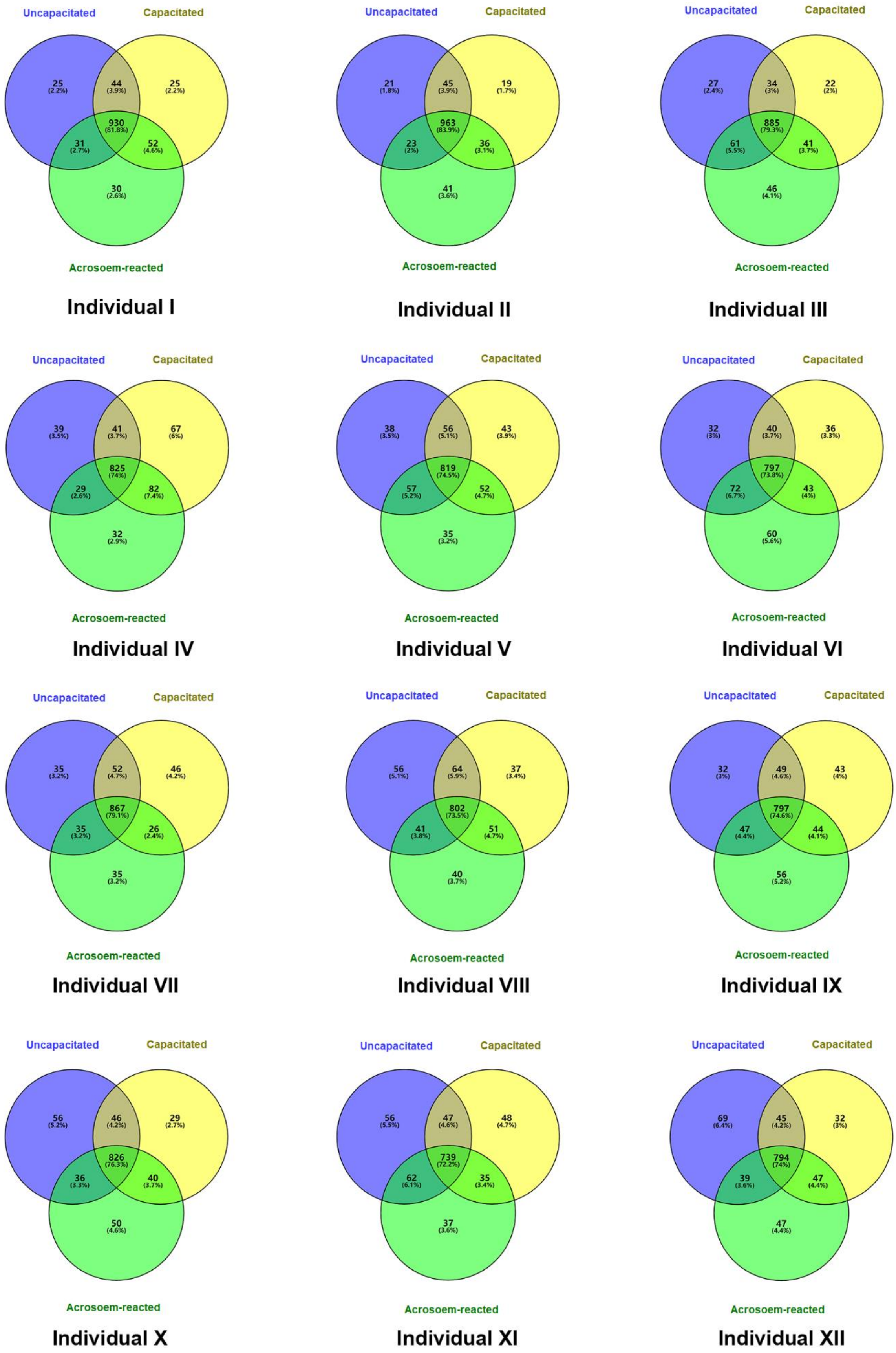

Supplementary Fig. 1. Venn diagram displaying the count of distinct lipids identified in spermatozoa sourced from multiple individuals under varying physiological states. Purple: Uncapacitated; Yellow: Capacitated; Green: Acrosome-reacted (induced by A23187).
